# A rare coexistence: Isolated prostate tuberculosis and nodular prostatic hyperplasia: A case report

**DOI:** 10.1016/j.ijscr.2025.111687

**Published:** 2025-07-15

**Authors:** Abebe Melis, Bizunesh Dires, Alemwosen Teklehaimanot, Gessesse Gesssesse, Teketel Tadesse

**Affiliations:** a Hawassa University College of Medicine and Health Science, Department of Pathology, Hawassa, Ethiopia; b Alatyon General Hospital, Hawassa, Ethiopia

**Keywords:** Prostate cancer, Prostate tuberculosis, Prostatic nodular hyperplasia, Case report

## Abstract

**Introduction and importance:**

Isolated prostate tuberculosis is exceedingly rare, accounting for only 2.6 % of genitourinary TB cases. This condition often presents with mildly elevated PSA levels and imaging findings that may resemble those of advanced prostate cancer. This case holds significant value due to the rarity of documented reports worldwide and diagnostic challenges. It provides an essential perspective for physicians, encouraging them to maintain a heightened index of suspicion for prostatic tuberculosis in patients presenting with vague lower urinary tract symptoms and features of anemia, particularly in endemic regions.

**Case presentation:**

Our case is a 75-year-old male patient who presented with non-specific lower urinary tract symptoms and features of anemia. Histopathologic examination confirmed the diagnosis of primary tuberculosis with nodular prostatic hyperplasia. Following anti-Tb treatment he showed notable improvement.

**Clinical discussion:**

The spread of infection to the prostate is primarily hematogenous. Prostatic tuberculosis is usually asymptomatic or subclinical in the early stage and nonspecific irritating micturition in the late stage. The mainstay of management for TB prostatitis is medical treatment using multiple anti-TB drug combinations. Surgical therapy can be considered if patients do not respond to medical therapy.

**Conclusion:**

Isolated prostatic tuberculosis is a rare clinical entity in which it can mimic non-specific prostatitis and prostatic carcinoma by its similar clinical presentation and digital rectal examination finding. Definitive diagnosis must be made by histopathological and bacteriologic studies.

## Highlights

• Isolated prostatic tuberculosis can clinically and radiologically mimic both prostate cancer and nodular prostatic hyperplasia, posing a diagnostic challenge.
• Histopathological evaluation revealing granulomatous inflammation with caseous necrosis is essential for definitive diagnosis in resource-limited, TB-endemic settings.
• Timely anti-TB therapy following surgical intervention led to full symptom resolution, highlighting the importance of early recognition and combined management.

## 1 Introduction

Tuberculosis (TB) is a significant global health concern caused by *Mycobacterium tuberculosis*. While it primarily affects the lungs, extrapulmonary involvement is also common, with the kidneys being the most frequently affected organ in the genitourinary system. Isolated prostate tuberculosis is exceedingly rare, accounting for only 2.6 % of genitourinary TB cases. This condition often presents with mildly elevated PSA levels and imaging findings that may resemble those of advanced prostate cancer [1]. PTB is a form of idiopathic infective granulomatous prostatitis, which often occurs in seminal vesicle tuberculosis and epididymal tuberculosis simultaneously. PTB is usually asymptomatic or subclinical in the early stage and nonspecific irritating micturition in the late stage. PTB is a serious and insidious disease and is often found accidentally in partial prostatectomy or biopsy. In PTB examination, prostatic hypertrophy, palpation with or without pain, and hard areas can be found by digital rectal examination. Meanwhile, PSA may be normal or elevated, and urine analysis and urine culture are usually negative [2]. Tuberculous prostatitis is often asymptomatic in the early stage, and imaging features are not well characterized in the literature, its diagnosis has been extremely challenging [2,3]. Patients with prostate tuberculosis may present asymptomatically or with nonspecific lower urinary tract symptoms. TB involving the prostate gland, apart from being rare, can also mimic cancer of the prostate as well as benign prostatic hyperplasia and therefore requires a high index of suspicion [2].

The work has been reported in line with the SCARE criteria [4].

## 2 Case presentation

Our case is a 75-year-old male patient who presented with 6 months history of pain during urination, urgency, frequency, hesitancy during urination and hematuria. Additionally, the patient experienced easy fatigability, tinnitus and vertigo. Otherwise, there was no history of trauma to the area or any other chronic illnesses. Notably, he exhibited no constitutional symptoms such as night sweats, fever, or signs of chronic illness, making clinical suspicion for tuberculosis less likely. He had no personal or family history of tuberculosis or prostate carcinoma. He has no history of treatment for STI or genital infection. He has no history of DM or HTN.

## 3 Physical findings

Vital signs were all within the normal limits.

Pertinent finding: Conjunctival paleness.

Enlarged prostate with firm texture and nodular surface on digital rectal examination.

Laboratory investigations revealed:

Full blood count (WBC – 4.8 × 10^9^/L, Hgb – 4.7 g/dL, MCV – 86.9 fL, platelet – 245 × 10^3^/L), liver and renal function tests were within the normal range. Antibody tests for HIV and VDRL infections were negative. Serum was PSA 15.5 ng/mL (Table 1).

**Table 1:** Summary of the patient's baseline laboratory investigations at initial presentation.

Parameters	Results	Normal range
WBC	4800/μL	3500−11,000/μL
Platelet	245 × 10^3^/μL	150−450 × 10^3^/μL
MCV	86.9 fL	80–100 fL
Hemoglobin	4.7 g/dL	12.5–16.3 g/dL
PSA	15.5 ng/mL	0–4 ng/mL
Creatinine	1.1 mg/dL	0.5–1.2 mg/dL
Urea	27 mg/dL	10-50 mg/dL
ALT	37I U/L	0–40 IU/L
AST	22I U/L	0–40 IU/L
HBV	Negative	Negative
HCV	Negative	Negative
PICT	Negative	Negative

Peripheral blood smears: Showed Normocytic Normochromic anemia.

On abdominopelvic ultrasound: Prostate size was 82 cc in volume and has internal heterogeneity with hypoechoic focus. Median lobe was prominent and protruded in the urinary bladder. Urinary bladder had circumferential diffuse wall thickening (4.6 mm) and internal echo-debris in the lumen with conclusion of enlarged prostate with bladder outlet obstruction and associated chronic cystitis.

For obstructive and irritative symptoms of the lower urinary tract secondary to benign prostatic hyperplasia and to rule out prostatic cancer, the patient underwent transvesical prostatectomy with smooth post-op condition.

**Fig. 1:**
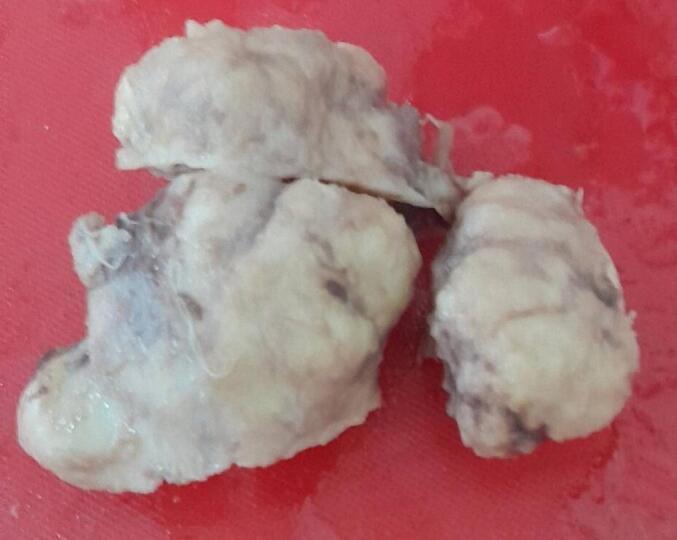
Gross image: enlarged prostatic tissues with nodular gray white outer surface.

**Fig. 2:**
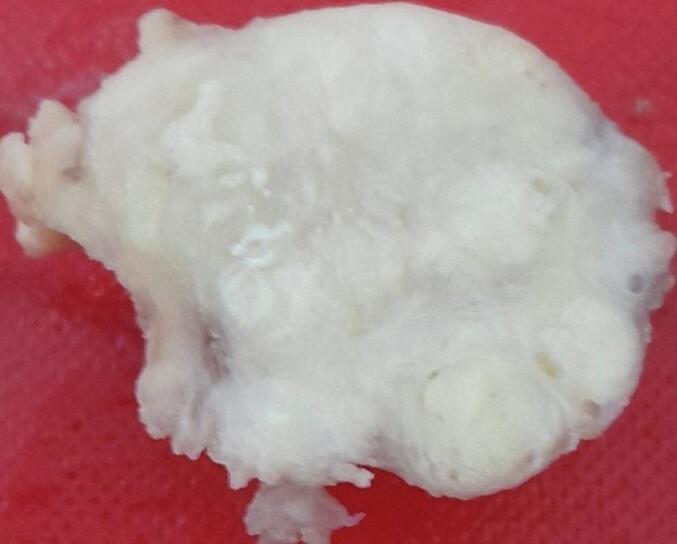
Cross-section revealing gray white nodular honeycombed tissue with foci of caseous necrosis.

**Fig. 3:**
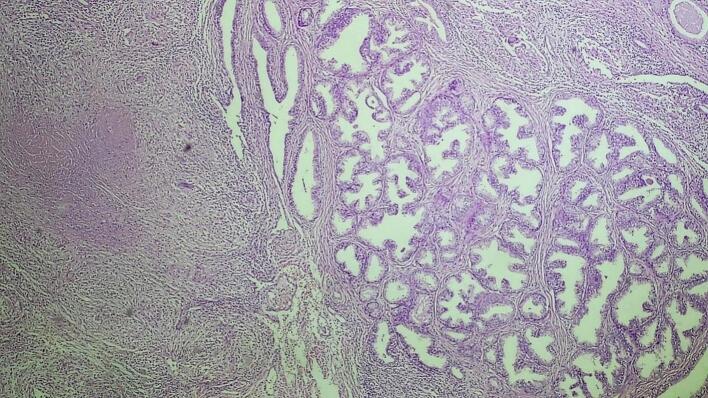
Low power microscopic examination demonstrating caseating granulomatous prostatitis with adjacent nodular hyperplasia.

**Fig. 4:**
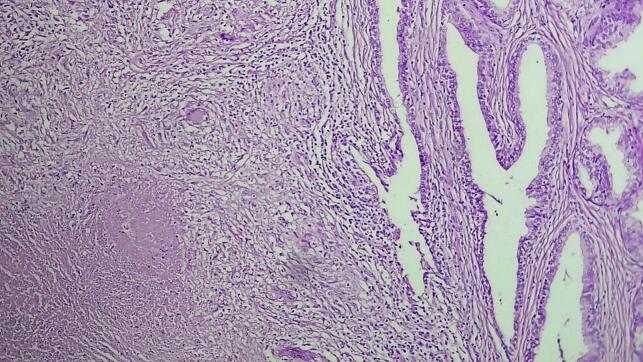
Medium power examination revealing caseous necrosis and langhans type multinucleated giant cells.

**Fig. 5:**
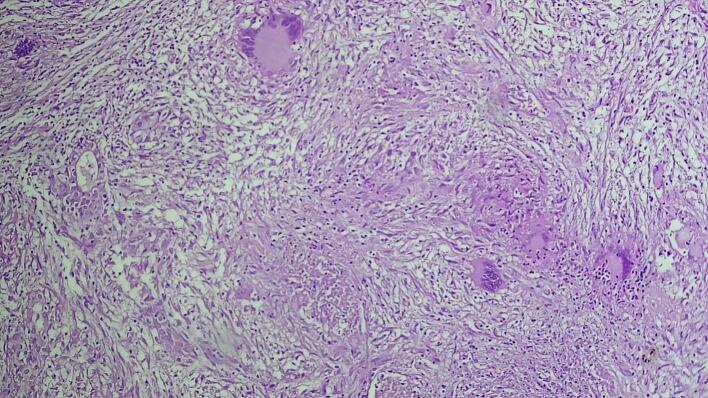
High power examination revealing caseous necrosis and langhans type multinucleated giant cells.

The excised prostate tissues appeared enlarged, measuring 7 × 5 × 2 cm and 3 × 2 × 2 cm having gray white nodular surface with varying consistency (Fig. 1). Cross-section revealed gray white nodular honeycombed tissue with foci of caseous necrosis (Fig. 2). No visible purulent material or distinct malignant features were noted. Hematoxylin and eosin-stained tissue microscopy showed nodular fibromuscular and glandular proliferation of prostatic tissue with mixed inflammatory infiltrates epitheloid cell granuloma, langhans type multinucleated giant cells and abundant caseous necrosis. No features of malignancy were noted (Fig. 3, Fig. 4, Fig. 5). Final histopathological diagnosis of prostatic tuberculosis with nodular prostatic hyperplasia was made.

The patient commenced anti-tuberculosis therapy for six months based on local guidelines. Combination therapy with rifampicin, isoniazid, pyrazinamide, and ethambutol was administered for two months, then the patient gets symptomatic reliefs including pain during urination, frequency and urgency symptoms, follow up PSA shows significant drop in number (8.5 ng/mL). Followed by isoniazid and rifampicin for four months. After completing antituberculosis therapy the patient showed a significant improvement with complete relief of the symptoms. After the completion of the treatment PSA comes within the normal range (3 ng/mL).

## 4 Discussion

Prostatic tuberculosis is a form of infective granulomatous prostatitis, which often occurs in seminal vesicle tuberculosis and epididymal tuberculosis simultaneously [5]. Pulmonary TB is the most common form of the disease and comprises 68.4 % of all cases. About 20 %–25 % of cases are extrapulmonary while only 27 % of the extrapulmonary TB involves the genitourinary system [6]. TB of the prostate gland is seen in only 2.6 % of genitourinary TB (GUTB) [1,7]. It's first described in 1882 by Jasmin [8]. The spread of infection to the prostate is primarily hematogenous, although lymphatic spread, descending infections from the upper urinary tract, direct intra-canalicular extension, and post-intravesical BCG injections are also documented pathways. This infection is promoted by some immunosuppressive diseases in developed countries such as AIDS and taking of corticosteroid and chemotherapy [9]. Prostatic tuberculosis is usually asymptomatic or subclinical in the early stage and nonspecific irritating micturition in the late stage. The clinical signs of lower urinary tract obstruction such as dysuria, urinary frequency and perineal heaviness can be observed but our patient presented with painless hematuria and non-specific symptoms of anemia [5]. Tuberculous prostatitis is a serious and insidious disease and is often found accidentally in partial prostatectomy or biopsy. In examination: prostatic hypertrophy, palpation with or without pain, and hard areas can be found by digital rectal examination [5].

However, Tuberculous prostatitis is difficult to distinguish from nonspecific prostatitis, benign prostatic hyperplasia, and Prostatic carcinoma, because they have similar clinical and imaging changes, resulting in difficult and delayed diagnosis [3]. Urine analysis may reveal sterile pyuria, which should help clinicians make a differential diagnosis of tuberculous prostatitis. The serum PSA level can be mildly elevated in TB prostatitis; however, extremely high levels are typically associated with prostate cancer. The mechanism for elevated PSA levels in prostate cancer is primarily attributed to increased production and release from cancerous prostate cells. In contrast, tuberculosis is characterized by inflammation and necrosis, and the mechanisms leading to high PSA levels in prostate tuberculosis remain largely unexplained [1]. Therefore, imaging manifestations of Prostatic tuberculosis should be more recognized, and early diagnosis should be improved. In prostatic carcinoma, because of the tight intercellular space connection and less water, the diffusion of water molecules is often limited or not diffused, and the apparent diffusion coefficient (ADC) signal is low. Imaging findings can also be misleading and may mimic prostate cancer; however, a discordance between a low PIRAD score and the presence of invasive features in a lesion in the peripheral zone is found to be suspicious for granulomatous prostatitis. Confirmatory diagnostic tests include histopathological examination and bacteriological evidence from culture [9]. More advanced and newer techniques for the diagnosis of prostate TB include cartridge-based nucleic acid amplification test (CBNAAT) of biopsy tissue and urine PCR. These techniques are applied when other diagnostic tests fail to detect mycobacteria [1].

The mainstay of management for TB prostatitis is medical treatment using multiple anti-TB drug combinations. Surgical therapy can be considered if patients do not respond to medical therapy or if a prostatic abscess is present, for which transurethral loop drainage is a feasible alternative option. PSA levels should be monitored in cases of elevated PSA to rule out an underlying prostate cancer [10]. In an extremely elevated PSA level, it was suggested to consider ongoing acute prostatitis as a possibility and repeat PSA level after treatment [11]. Delays in diagnosis can lead to complications such as abscess formation, urethral stricture, and systemic dissemination of the disease, all of which may significantly impact a patient's quality of life and reproductive health [9].

## 5 Conclusions

Isolated prostatic tuberculosis and nodular prostatic hyperplasia may co-exist in some cases and clinicians must have a high index of suspicion for tuberculosis, especially in patients from endemic areas, in order to initiate early and proper treatment. Definitive diagnosis must be made by histopathological and bacteriologic studies. Once its diagnosed multiple anti-TB drug combination is the mainstay of management.

## Author contribution

Dr. Abebe Melis Nisro, MD - Study concept and design, writing the paper, literature review and editing and critical review of the paper.

Dr. Bizunesh Dires and Dr. Alemwosen T/haimanot, MD - Study concept, writing the paper, data interpretation and critical review of the paper.

Dr. Gessesse Gesssesse, MD – Study concept, acquisition of data, literature review of the paper, editing and critical review of the paper.

Dr. Teketel Tadesse, MD - Study concept and design, writing the paper, literature review and editing and critical review of the paper.

## Informed consent

The patient provided written informed consent in their native language for the publication of non-identifying information, including intraoperative images. A copy of the consent document can be reviewed by the Editor-in-Chief of this journal upon request.

## Ethical approval

According to the ethical committee of Hawassa University College of Medical and Health Sciences, formal ethical approval is not required for this case, as it represents a single occurrence observed in practice and does not involve experiments on humans or animals.

## Guarantor

Abebe Melis Nisro, MD.

## Research registration number

Not applicable.

## Funding

No funding was used in this study.

## Conflict of interest statement

No conflicts of interest.

## Data Availability

The data used to support the findings of this case report are available from the corresponding author upon reasonable request.

## References

[1] Tesfaye Kebede Legesse, Semira Abrar Issa, Yodit Abraham Yaynishet, Tesfahun Amsal Dessie, Tewodros Yalew Gebremariam, Birhanu Kassie Reta (2024). Isolated prostate tuberculosis mimicking prostate cancer. Ethiop. J. Health Sci..

[2] Mishra K.G., Ahmad A., Singh G., Tiwari R. (2019). Tuberculosis of the prostate gland masquerading prostate cancer; five cases experience at IGIMS. Urol. Ann..

[3] Li Y., Dan S., Yang F., He X., He L., Yue W. (Nov 24 2023). Prostate tuberculosis mimicking prostate cancer: case report and literature review. Medicine (Baltimore).

[4] Kerwan A., Al-Jabir A., Mathew G., Sohrabi C., Rashid R., Franchi T., Nicola M., Agha M., Agha R.A. (2025). Revised surgical CAse REport (SCARE) guideline: an update for the age of artificial intelligence. Premier J. Sci..

[5] Aziz E.M., Abdelhak K., Hassan F.M. (2016). Tuberculous prostatitis: mimicking a cancer. Pan Afr. Med. J..

[6] Chan W.C., Thomas M. (2000). Prostatic abscess: another manifestation of tuberculosis in HIV-infected patients. Aust. NZ J. Med..

[7] Kulchavenya E., Brizhatyuk E., Khomyakov V. (2014). Diagnosis and therapy for prostate tuberculosis. Ther. Adv. Urol..

[8] Benchekroun A., Iken A., Qarro A., Aelalj H., Nouini Y., Benslimane L. (2003). La tuberculose prostatique: à propos de 2 cas. Ann. Urol..

[9] Figueiredo A.A., Lopes H.E., Barreto A.A., Fanni V.S.S., Bastos J.M. Netto (Jan-Feb 2024). Prostate tuberculosis: six forms of clinical presentation. Int. Braz. J. Urol..

[10] Tapsoba A.K., Rahoui M., Bibi M., Chelly B., Ouanes Y., Chaker K. (2021). An unusual association of adenocarcinoma and isolated tuberculosis of the prostate gland. J. Surg. Case Rep..

[11] Nepal A., Sharma P., Bhattarai S., Mahajan Z., Sharma A., Sapkota A. (2023). Extremely elevated prostate-specific antigen in acute prostatitis: a case report. Cureus.

